# Association between controlling nutritional status score and the prognosis of patients with acute myocardial infarction: a systematic review and meta-analysis

**DOI:** 10.3389/fnut.2024.1518822

**Published:** 2025-01-15

**Authors:** Lei Peng, Jian Tang, Ningjun Zhang, Zhongnan Zhang, Deqi Wang, Youfu He

**Affiliations:** ^1^Department of Cardiology, Linping Hospital of Integrated Traditional Chinese and Western Medicine, Hangzhou, China; ^2^Department of Cardiology, Beijing University of Chinese Medicine East Hospital, Zaozhuang Hospital, Zaozhuang, China; ^3^Zhejiang University School of Medicine, Jinhua, China; ^4^Department of Interventional Cardiology, Zaozhuang Municipal Hospital, Zaozhuang, China; ^5^Department of Cardiology, Guizhou Provincial People’s Hospital, Guiyang, China

**Keywords:** controlling nutritional status, acute myocardial infarction, prognosis, nutritional risk, systematic review

## Abstract

**Background:**

Recent studies have reported growing evidence supporting applying the controlling nutritional status (CONUT) score in acute myocardial infarction (AMI) patients. This investigation intended to ascertain the link between CONUT scores and the prognosis in the AMI population.

**Methods:**

Multiple electronic databases, encompassing PubMed, Web of Science, Embase, and the Cochrane Library, were retrieved from the inception of the databases until July 20, 2024, to explore the link between CONUT scores and adverse clinical outcomes in individuals with AMI. Primary outcomes consisted of major adverse cardiovascular events (MACE) and mortality, while secondary outcomes encompassed stroke, cardiac death, myocardial reinfarction, revascularization, ventricular arrhythmias, and atrioventricular block. A random-effects meta-analysis was executed, with CONUT scores treated as either categorical or continuous variables. Sensitivity analyses and Egger’s test were conducted to appraise the robustness of results and publication bias, respectively. Subgroup analyses were executed to account for various confounding factors. Moreover, the GRADE system was leveraged to appraise the quality of evidence for all outcomes.

**Results:**

Fifteen studies were included in our analysis. The statistical analyses on both categorical and continuous variables unraveled that a high CONUT score was markedly linked to an elevated risk of MACE [categorical variable: odds ratio (OR) = 1.75, 95% confidence interval (CI) = 1.42–2.15; continuous variable: standardized mean difference (SMD) = 1.02, 95% CI = 0.78–1.26], mortality (categorical variable: OR = 2.08, 95% CI = 1.70–2.55; continuous variable: SMD = 1.16, 95% CI = 0.57–1.74), cardiac death (categorical variable: OR = 2.81, 95% CI = 1.67–4.73), myocardial reinfarction (categorical variable: OR = 2.21, 95% CI = 1.28–3.83), and atrioventricular block (categorical variable: OR = 5.21, 95% CI = 1.83–14.89) in AMI patients. However, no significant association was found between a high CONUT score and stroke (categorical variable: OR = 1.52, 95% CI = 0.98–2.35), revascularization (categorical variable: OR = 2.92, 95% CI = 0.58–14.79), and ventricular arrhythmias (categorical variable: OR = 2.57, 95% CI = 0.06–107.21).

**Conclusion:**

The CONUT score may serve as a promising and cost-effective prognostic biomarker for individuals with AMI.

**Systematic review registration:**

PROSPERO: CRD42024574048.

## 1 Introduction

Acute myocardial infarction (AMI) represents a leading contributor to death across the world (1). With advances in early reperfusion strategies, pharmacological treatments, and standardized care, the prognosis of AMI has significantly improved over the past few decades (2–4). However, despite early treatment, some individuals still face risks of mortality and major adverse cardiovascular events (MACE). Even in modern times, the 10-year mortality and readmission rates for recurrent myocardial infarction patients hospitalized for 30 days due to AMI in the United States exceed 70 and 25%, respectively (4). Therefore, selecting appropriate preventive strategies and identifying risk factors contributing to adverse outcomes in AMI patients are of paramount importance.

In recent years, studies have shown that the risk of malnutrition is linked to elevated in-hospital mortality, mid- and long-term mortality, and cardiovascular events in patients with cardiovascular diseases (5, 6). Additionally, the relationship between the CONUT score and stroke or other diseases has also been reported in detailed systematic reviews (7). Currently, various tools have been created to determine the nutritional status of hospitalized patients (8). Among them, the CONUT score is a newly emerged tool for appraising nutritional status. It is an immunonutritional index computed based on serum albumin concentration, total lymphocyte count, and total cholesterol concentration (9), which can evaluate protein reserves, lipid metabolism, and immune defense. Evidence demonstrates that the CONUT score performs well in forecasting the progression and clinical outcomes of cardiovascular diseases. Additionally, previous studies have demonstrated that the CONUT score is closely associated with inflammatory responses (10) and immune function (11). A higher CONUT score is often linked to stronger inflammatory responses and an immunosuppressive state (12, 13), suggesting that patients may be at risk of malnutrition or immune dysfunction. Assessing the degree of malnutrition risk through the CONUT score can indirectly reflect the body’s inflammatory burden and immune response levels, thus providing valuable diagnostic and therapeutic insights for clinical practice (14). Considering the strong correlations among cardiovascular diseases, nutritional status, inflammation, and immune responses, increasing evidence has highlighted the significant association between the CONUT score and the progression and clinical outcomes of cardiovascular diseases. For example, Lu et al. (15) found that the risk of malnutrition was independently linked to an elevated risk of all-cause mortality in critically ill AMI patients based on a cohort study that included 2,962 AMI patients from Chinese and U.S. databases. Another study has unveiled that higher CONUT scores are linked to an elevated risk of MACE in patients with ST-segment elevation myocardial infarction (STEMI), complications in patients with acute coronary syndrome (ACS), as well as complications and mortality in individuals receiving coronary artery bypass grafting (16). Additionally, poor nutritional status after AMI has been linked to a higher likelihood of experiencing myocardial reinfarction, stroke, revascularization, ventricular arrhythmias, and cardiovascular death (17). Nonetheless, some studies have raised concerns about the efficiency of the CONUT score in forecasting AMI prognosis. For instance, Zhu et al. (18) utilized the CONUT score and the prognostic nutritional index (PNI) as nutritional status indicators and observed that PNI was a more reliable index for forecasting MACE in ACS patients compared to CONUT score.

A recent meta-analysis regarding the prognostic impact of the CONUT score in patients with coronary artery disease (CAD) has been published (19). Nevertheless, it primarily focused on CAD patients, and only incorporated limited reports on AMI. To date, no MA has systematically elucidated the influence of the CONUT score on prognostic outcomes specifically in AMI patients. Consequently, this meta-analysis intended to probe into the precise predictive efficiency of the CONUT score for the prognosis of AMI.

## 2 Materials and methods

### 2.1 Protocol registration

The current meta-analysis was registered with the International Prospective Register of Systematic Reviews (PROSPERO) (CRD42024574048). There were no deviations from the study protocol. The current study was conducted as per the guidelines of the Preferred Reporting Items for Systematic Reviews and Meta-Analyses (PRISMA) (20).

### 2.2 Study selection

The inclusion criteria were designed as follows:

P: The study population consisted of AMI patients (including those with acute STEMI or NSTEMI).
E: High CONUT score.
C: Low CONUT score.
O: Clinical outcomes of AMI, including MACE, mortality, stroke, cardiac death, myocardial reinfarction, revascularization, ventricular arrhythmias, and atrioventricular block.
S: Randomized controlled trials, cohort studies, and case-control studies.

The following studies were excluded:

1. Case reports, reviews, letters, conference abstracts, and commentaries.
2. Animal studies.
3. Studies with duplicate or overlapping data.
4. Non-English literature.

### 2.3 Literature search

An array of electronic databases, including PubMed, Web of Science, Embase, and the Cochrane Library, were comprehensively searched from their inception until July 20, 2024. The subject headings encompassed *Controlling Nutritional Status*, *CONUT*, and *myocardial infarction*. The search strategy for PubMed was listed as follows: ((Controlling Nutritional Status) OR (CONUT)) AND ((“Myocardial Infarction”[Mesh]) OR (((((((((((((Infarction, Myocardial) OR (Infarctions, Myocardial)) OR (Myocardial Infarctions)) OR (Heart Attack)) OR (Heart Attacks)) OR (Myocardial Infarct)) OR (Infarct, Myocardial)) OR (Infarcts, Myocardial)) OR (Myocardial Infarcts)) OR (Cardiovascular Stroke)) OR (Cardiovascular Strokes)) OR (Stroke, Cardiovascular)) OR (Strokes, Cardiovascular))). The detailed search strategies for the other databases are depicted in Supplementary Table S1.

### 2.4 Data extraction

Two independent researchers (LP and YH) appraised the quality of the eligible studies and extracted data from the eligible research articles. Any disagreements were discussed with all co-authors to reach a consensus. The extracted data encompassed the first author’s name, publication year, country, study population, study duration, timing of assessment, treatment methods, sample size, age, gender, body mass index (BMI), follow-up period, cutoff values, as well as OR, RR, or HR with 95% CI, and SMD. The primary outcome measures were MACE and mortality in AMI patients, while secondary outcome measures included stroke, cardiac death, myocardial reinfarction, revascularization, ventricular arrhythmias, and atrioventricular block.

### 2.5 Quality assessment

The Newcastle–Ottawa Scale (NOS) was leveraged by two reviewers (LP and JT) to independently appraise the quality of eligible studies (21). The NOS tool consisted of three domains: selection (4 points), comparability (2 points), and outcome assessment and adequacy of follow-up (3 points). The total NOS score ranged between 0 and 9, and a score of ≥7 was indicative of high quality.

### 2.6 Statistical analysis

To estimate the prognostic impact of the CONUT score in AMI patients, the effect sizes and 95% CI were pooled leveraging a random-effects model. Categorical data were combined utilizing OR, while continuous data were combined utilizing SMD. Cochran’s *Q* test and Higgins’ *I*^2^ statistics were leveraged to determine heterogeneity across studies. An *I*^2^ value >50% or *p* < 0.1 indicated considerable heterogeneity. Sensitivity analyses and subgroup analyses by various factors were performed to check the reliability of the CONUT score in predicting AMI outcomes and to determine the heterogeneity sources. Besides, Egger’s test and funnel plots were utilized to clarify publication bias. A *p*-value of <0.05 was indicative of statistical significance. STATA 15.0 and Review Manager 5.4 software were adopted to carry out statistical analyses. Additionally, the quality of evidence for all outcomes was evaluated utilizing the GRADE system. The evidence quality was rated as very low, low, moderate, or high (22).

## 3 Results

### 3.1 Characteristics of eligible studies

**Figure 1:**
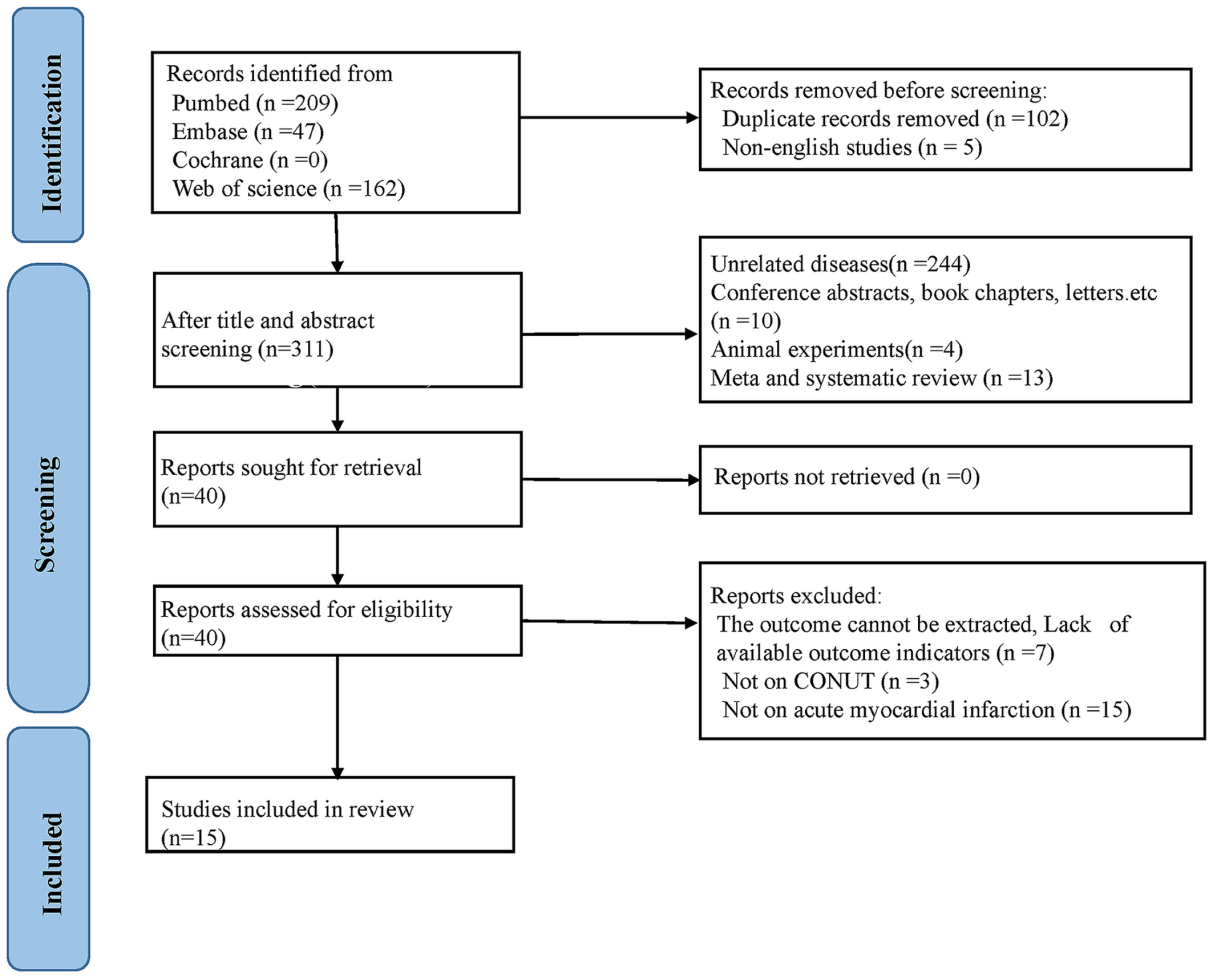
Flowchart of literature screening.

Four hundred and eighteen records were initially retrieved from the four major databases. After deleting 102 duplicate articles, five non-English studies, and four animal experiments, 267 studies were deleted after checking the titles and abstracts of the remaining studies. Subsequently, the full texts of the remaining 40 studies were reviewed. Among them, 25 publications were further removed since they were not related to AMI (*n* = 15), not related to CONUT score (*n* = 3), or lacked outcome measures (*n* = 7). Ultimately, 15 studies were incorporated into this analysis (15–17, 23–34) (Figure 1).

The sample sizes of these eligible studies varied from 86 to 4,525 patients, with a cumulative sample size of 14,302 patients. Four studies (15, 28, 31, 32) were conducted in China, four (16, 30, 33, 34) in Turkey, two (17, 26) in Romania, and one each in Spain (23), Japan (24), the United States (15), Singapore (25), India (27), and Italy (29). One study (15) included two cohort studies from different countries, both of which encompassed three prognostic indicators for mild, moderate, and severe malnutrition, and was thus classified as six independent studies. Similarly, one study (23) with three prognostic indicators for mild, moderate, and severe malnutrition, was classified as three independent studies, while one study (32), with two prognostic indicators for mild and moderate malnutrition, was classified as two independent studies. Consequently, 23 studies were finally included. Among them, 20 were retrospective studies (15, 16, 23–25, 28–34), and three were prospective studies (17, 26, 27). These studies were published between 2016 and 2024. Of the 23 studies, 23 provided dichotomous outcome data on the link between the CONUT score and AMI prognosis (15–17, 23–34), while five studies (24, 27, 29, 31, 34) reported continuous variable data. The detailed baseline features of the eligible publications are presented in Table 1.

**Table 1:** Basic characteristics of included studies.

ID	Author	Years	Study period	Region	Study design	Population	Time of test	Treatment	Follow-up	No. of patients	Gender		Mean/median age	BMI (kg/m^2^)	Cut off
											Male	Female			
1	Zengin et al.	2022	2014–2017	Turkey	Cohort studies	STEMI	Before treatment	PCI	19.9 months	1,028	772	256	58	NA	5
2	Roubín et al. (a)	2020	2010–2017	Spain	Cohort studies	AMI	Before treatment	PCI or medical management	3.6 years	4,525	NA	NA	66.2	27.97	NA
2	Roubín et al. (b)	2020	2010–2017	Spain	Cohort studies	AMI	Before treatment	PCI or medical management	3.6 years	4,525	NA	NA	66.2	27.97	NA
2	Roubín et al. (c)	2020	2010–2017	Spain	Cohort studies	AMI	Before treatment	PCI or medical management	3.6 years	4,525	NA	NA	66.2	27.97	NA
3	Takahashi et al.	2021	2013–2017	Japan	Cohort studies	AMI	Before treatment	PCI	1,234 days	186	NA	NA	68.4	23.8	3
4	Lu et al. (a)	2022	2007–2018	China	Cohort studies	AMI	Before treatment	PCI or CAG	4 years	2075	1,660	415	62.5	NA	4
4	Lu et al. (b)	2022	2007–2018	China	Cohort studies	AMI	Before treatment	PCI or CAG	4 years	2075	1,660	415	62.5	NA	4
4	Lu et al. (c)	2022	2007–2018	China	Cohort studies	AMI	Before treatment	PCI or CAG	4 years	2075	1,660	415	62.5	NA	4
4	Lu et al. (d)	2022	2001–2012	USA	Cohort studies	AMI	Before treatment	Medical management	4 years	887	555	332	70.1	NA	4
4	Lu et al. (e)	2022	2001–2012	USA	Cohort studies	AMI	Before treatment	Medical management	4 years	887	555	332	70.1	NA	4
4	Lu et al. (f)	2022	2001–2012	USA	Cohort studies	AMI	Before treatment	Medical management	4 years	887	555	332	70.1	NA	4
5	Kong et al.	2023	2014–2021	Singapore	Cohort studies	AMI	Before treatment	PCI	2 years	1829	1,384	445	66	25	2
6	Czinege et al.	2024	2023.3.1–2023.5.15	Romania	Cohort studies	AMI	Before treatment	PCI	3 months	108	NA	NA	61.62	24.24	3
7	Rus et al.	2020	2018.1.1–2018.2.28	Romania	Cohort studies	AMI	Before treatment	PCI	5 days	86	54	32	61.36	28.46	3
8	Mangalesh et al.	2023	2021–2022	India	Cohort studies	STEMI	Before treatment	PCI	28 days	402	260	142	75.0	24.35	4
9	Ni et al.	2023	2013–2021	China	Cohort studies	STEMI	Before treatment	PCI	31 months	442	NA	NA	73	23.41	NA
10	Basta et al.	2016	2006–2012	Italy	Cohort studies	STEMI	Before treatment	PCI	2 years	945	705	240	65.7	27	NA
11	Boyraz et al.	2022	2017–2020	Turkey	Cohort studies	NSTEMI	Before treatment	PCI	6 months	205	103	102	74.49	NA	3
12	Chen et al.	2020	2014–2017	China	Cohort studies	AMI	Before treatment	PCI	2 years	107	86	21	72	22.39	3.5
13	Deng et al. (a)	2020	2015–2018	China	Cohort studies	STEMI	Before treatment	PCI	24.6 months	751	621	130	64.0	NA	5
13	Deng et al. (b)	2020	2015–2018	China	Cohort studies	STEMI	Before treatment	PCI	24.6 months	751	621	130	64.0	NA	5
14	Kalyoncuoğlu et al.	2021	2017–2019	Turkey	Cohort studies	NSTEMI	Before treatment	PCI	20.5 months	253	181	72	68.5	28	2
15	Yıldırım et al.	2021	2014–2015	Turkey	Cohort studies	NSTEMI	Before treatment	PCI	64.5 months	915	471	444	73.1	25.22	3

STEMI, ST-segment elevation myocardial infarction; NSTEMI, non-ST segment elevation myocardial infarction; CAG, coronary angiography; PCI, percutaneous coronary intervention; AMI, acute myocardial infarction; BMI, body mass index.

### 3.2 Study quality

The NOS scores of the eligible publications varied from 7 to 8, unveiling that they were of high quality. The specific NOS scores are provided in Supplementary Table S2.

### 3.3 Meta-analysis results

#### 3.3.1 Association between CONUT score and MACE

**Figure 2:**
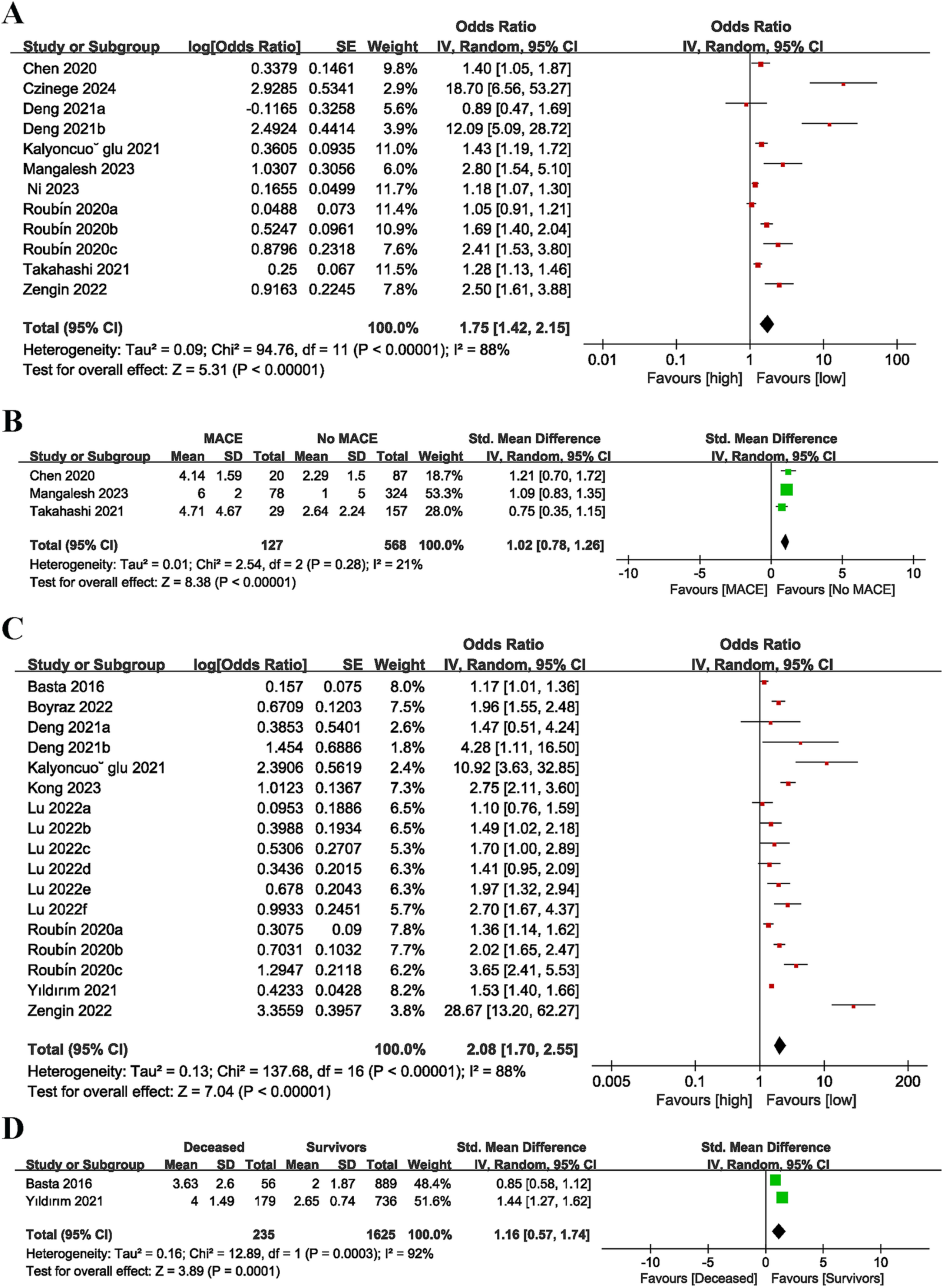
Forest plots for the association between CONUT score and MACE (**A**: categorical variable; **B**: continuous variable) and the association between CONUT score and mortality (**C**: categorical variable; **D**: continuous variable).

Twelve studies (16, 17, 23, 24, 27, 28, 31–33) reported MACE outcomes using CONUT score as a categorical variable. Considerable heterogeneity was detected (*I*^2^ = 88%, *p* < 0.00001). The pooled result (OR = 1.75, 95% CI = 1.42–2.15, *p* < 0.00001) unraveled that an elevated CONUT score was markedly linked to an elevated risk of MACE in the AMI population (Figure 2A).

Three studies (24, 27, 31) utilized CONUT score as a continuous variable. No considerable heterogeneity was noted (*I*^2^ = 21%, *p* = 0.28). The pooled result (OR = 1.02, 95% CI = 0.78–1.26, *p* < 0.00001) unraveled that individuals with AMI patients who developed MACE had considerably elevated CONUT score values in comparison to those who did not experience MACE (Figure 2B).

#### 3.3.2 Association between CONUT score and mortality

Seventeen studies (15, 16, 23, 25, 29, 30, 32–34) evaluated the link between CONUT score as a categorical variable and mortality. Considerable heterogeneity was noted (*I*^2^ = 88%, *p* < 0.00001). The pooled result (OR = 2.08, 95% CI = 1.70–2.55, *p* < 0.00001) indicated that a heightened CONUT score was markedly linked to a heightened risk of mortality in AMI patients (Figure 2C).

Two studies (29, 34) utilized CONUT score as a continuous variable. There was considerable heterogeneity (*I*^2^ = 92%, *p* = 0.0003). The pooled result (OR = 1.16, 95% CI = 0.57–1.74, *p* = 0.0001) suggested that a heightened CONUT score was markedly linked to a heightened risk of mortality in individuals with AMI (Figure 2D).

#### 3.3.3 Association between CONUT score and stroke

**Figure 3:**
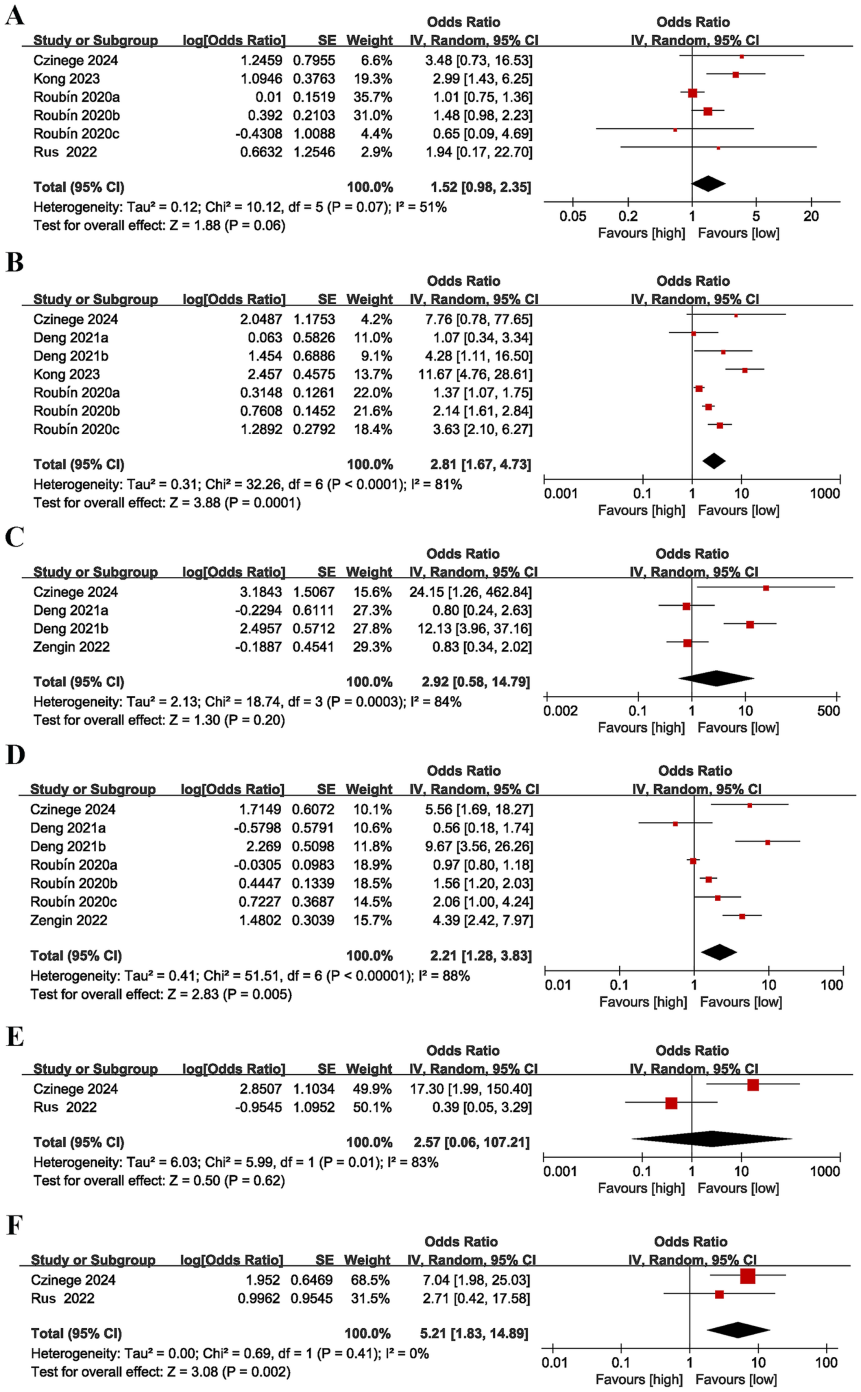
Forest plots for the association between CONUT score and various outcomes: **(A)** stroke (categorical variable), **(B)** cardiac death (categorical variable), **(C)** revascularization (categorical variable), **(D)** myocardial reinfarction (categorical variable), **(E)** ventricular arrhythmias (categorical variable), **(F)** atrioventricular block (categorical variable).

Six studies (17, 23, 25, 26) reported the association between CONUT score as a categorical variable and stroke. Moderate heterogeneity was detected (*I*^2^ = 51%, *p* = 0.07). The pooled result (OR = 1.52, 95% CI = 0.98–2.35, *p* = 0.06) revealed no marked link between high CONUT scores and the risk of stroke (Figure 3A).

#### 3.3.4 Association between CONUT score and cardiac death

Seven studies (17, 23, 25, 32) evaluated the relationship between CONUT score as a categorical variable and cardiac death. There was considerable heterogeneity (*I*^2^ = 81%, *p* < 0.0001). The pooled result (OR = 2.81, 95% CI = 1.67–4.73, *p* = 0.0001) unveiled that a heightened CONUT score was markedly linked to an elevated risk of cardiac death (Figure 3B).

#### 3.3.5 Association between CONUT score and revascularization

Four studies (16, 17, 32) reported the association between CONUT score as a categorical variable and revascularization. Considerable heterogeneity was found (*I*^2^ = 84%, *p* = 0.0003). The pooled result (OR = 2.92, 95% CI = 0.58–14.79, *p* = 0.20) indicated no link between a heightened CONUT score and the risk of revascularization (Figure 3C).

#### 3.3.6 Association between CONUT score and myocardial reinfarction

Seven studies (16, 17, 23, 32) evaluated the relationship between CONUT score as a categorical variable and myocardial reinfarction. Considerable heterogeneity was found (*I*^2^ = 88%, *p* < 0.00001). The pooled result (OR = 2.21, 95% CI = 1.28–3.83, *p* = 0.005) suggested that a higher CONUT score was markedly linked to an elevated risk of myocardial reinfarction (Figure 3D).

#### 3.3.7 Association between CONUT score and ventricular arrhythmias

Two studies (17, 26) assessed the relationship between CONUT score as a categorical variable and ventricular arrhythmias. Considerable heterogeneity was detected (*I*^2^ = 83%, *p* = 0.01). The pooled result (OR = 2.57, 95% CI = 0.06–107.21, *p* = 0.62) unraveled no significant link between a heightened CONUT score and the risk of ventricular arrhythmias (Figure 3E).

#### 3.3.8 Association between CONUT score and atrioventricular block

Two studies (17, 26) evaluated the relationship between CONUT score as a categorical variable and atrioventricular block. No heterogeneity was noted (*I*^2^ = 0%, *p* = 0.41). The pooled result (OR = 5.21, 95% CI = 1.83–14.89, *p* = 0.002) indicated that a higher CONUT score was markedly linked to an elevated likelihood of developing atrioventricular block (Figure 3F).

### 3.4 Sensitivity analysis

**Figure 4:**
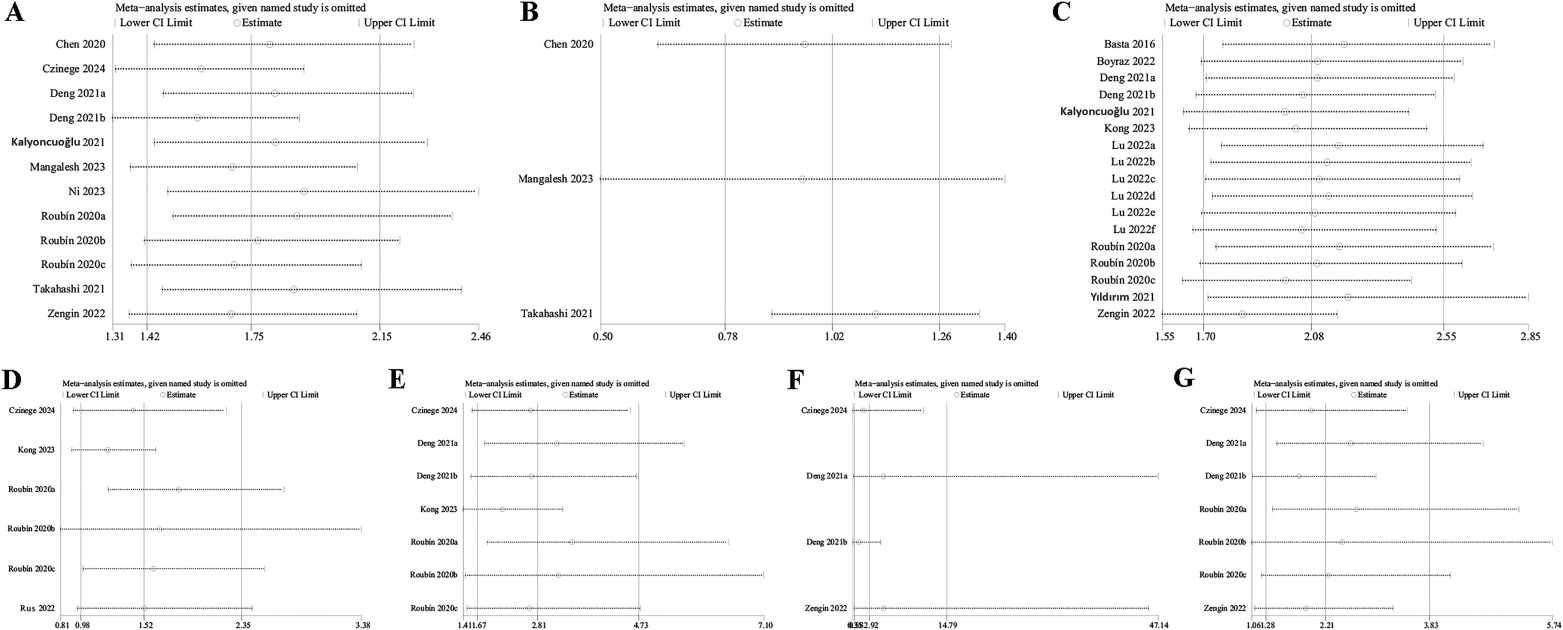
Sensitivity analyses of the association between CONUT score and different outcomes: **(A)** CONUT score and MACE as a categorical variable, **(B)** CONUT score and MACE as a continuous variable, **(C)** CONUT score and mortality as a categorical variable, **(D)** CONUT score and stroke as a categorical variable, **(E)** CONUT score and cardiac death as a categorical variable, **(F)** CONUT score and revascularization as a categorical variable, **(G)** CONUT score and myocardial reinfarction as a categorical variable.

Sensitivity analysis unraveled that all studies did not considerably influence the pooled results for MACE, mortality, cardiac death, myocardial reinfarction, or revascularization in AMI patients. The results of the categorical variable analysis for stroke were influenced by one included study. When we removed the study by Roubín 2020a, the pooled result changed to (OR = 1.82, 95% CI = 1.22–2.72, *p* = 0.003), and the heterogeneity considerably decreased (*I*^2^ = 8%, *p* = 0.36), suggesting the instability of the results for this outcome. Owing to a small number of studies on ventricular arrhythmias and atrioventricular block (*n* = 2), sensitivity analyses were not conducted for these outcomes. Detailed results are shown in Figures 4A–G.

### 3.5 Publication bias

**Figure 5:**
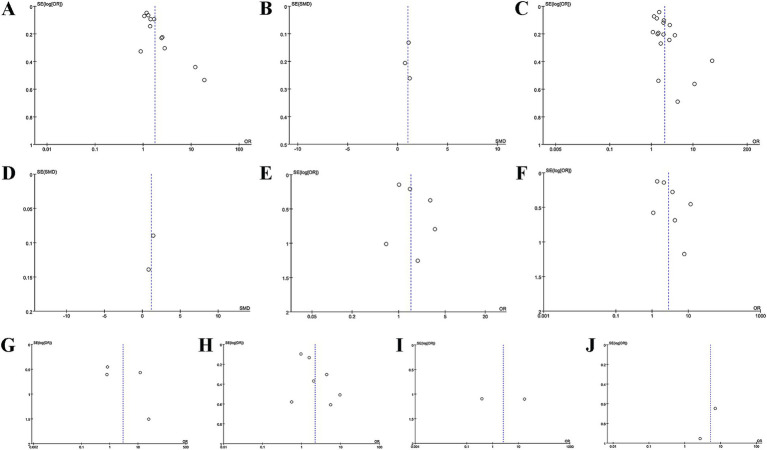
Funnel plots for assessing publication bias: **(A)** CONUT score and MACE as a categorical variable, **(B)** CONUT score and MACE as a continuous variable, **(C)** CONUT score and mortality as a categorical variable, **(D)** CONUT score and mortality as a continuous variable, **(E)** CONUT score and stroke as a categorical variable, **(F)** CONUT score and cardiac death as a categorical variable, **(G)** CONUT score and revascularization as a categorical variable, **(H)** CONUT score and myocardial reinfarction as a categorical variable, **(I)** CONUT score and ventricular arrhythmias as a categorical variable, **(J)** CONUT score and atrioventricular block as a categorical variable.

Funnel plots and Egger’s test were leveraged to ascertain publication bias. As shown in Figures 5A–J, the funnel plots were asymmetrical for MACE and mortality (categorical variables) and symmetrical for the remaining outcome measures. The results of Egger’s test were as follows: *p* = 0.003 for MACE (categorical variable), *p* = 0.895 for MACE (continuous variable), *p* = 0.023 for mortality (categorical variable), *p* = 1.09 for stroke (categorical variable), *p* = 0.157 for cardiac death (categorical variable), *p* = 0.511 for revascularization (categorical variable), *p* = 0.098 for myocardial reinfarction (categorical variable).

### 3.6 Subgroup analysis

The results of all subgroup analyses are shown in Table 2. For CONUT score (categorical variable), the results of subgroup analyses of MACE by population characteristics, follow-up duration, region or country, sample size, age, and cutoff values were all significant (all *p* < 0.05). When the studies stratified by treatment methods, the CONUT score was markedly efficient in predicting outcomes in AMI patients in the percutaneous coronary intervention (PCI) treatment group, but not in the mixed treatment group.

**Table 2:** Subgroup analysis of CONUT score and MACE and mortality in patients with AMI.

Subgroup	MACE (categorical variable)				Mortality (categorical variable)			
	Study	OR [95% CI]	*p*-value	*I* ^2^	Study	OR [95% CI]	*p*-value	*I* ^2^
Total population	12	1.75 [1.42–2.75]	0.00001	88%	17	2.08 [1.70–2.55]	0.00001	88%
STEMI	5	2.29 [1.18–4.45]	0.01	91%	4	3.78 [0.69–20.56]	0.12	95%
NSTEMI	1	1.43 [1.19–1.72]	0.0001	NA	3	2.14 [1.38–3.32]	0.0007	87%
Mixed	6	1.69 [1.26–2.27]	0.0005	90%	10	1.87 [1.50–2.35]	0.00001	80%
Follow-up								
>2 years	7	1.50 [1.19–1.89]	0.0006	89%	12	1.74 [1.48–2.06]	0.00001	70%
≤2 years	5	2.45 [1.51–3.96]	0.0003	87%	5	3.88 [1.96–7.69]	0.0001	96%
Region								
Asia	8	1.63 [1.30–2.05]	0.0001	85%	10	2.48 [1.74–3.54]	0.00001	90%
Europe	4	2.29 [1.31–3.99]	0.004	94%	4	1.77 [1.23–2.56]	0.002	92%
America					3	1.92 [1.34–2.74]	0.0003	53%
No. of patients								
>1,000	4	1.74 [1.15–2.61]	0.008	90%	8	2.39 [1.61–3.55]	0.0001	92%
≤1,000	8	1.81 [1.37–2.38]	0.0001	89%	9	1.80 [1.44–2.25]	0.00001	79%
Mean/median age								
>65 years	8	1.42 [1.22–1.64]	0.00001	80%	11	1.95 [1.60–2.37]	0.00001	87%
≤65 years	4	4.47 [1.33–15.01]	0.02	92%	6	2.70 [1.18–6.17]	0.02	91%
Cut-off								
>3	5	2.38 [1.27–4.47]	0.007	87%	9	2.33 [1.44–3.79]	0.0006	87%
≤3	3	2.02 [1.22–3.33]	0.006	92%	4	2.34 [1.57–3.50]	0.0001	90%
Treatment								
PCI only	9	1.89 [1.45–2.47]	0.00001	89%	8	2.76 [1.89–4.03]	0.00001	93%
Mixed	3	1.56 [1.01–2.41]	0.05	91%	9	1.78 [1.42–2.23]	0.00001	76%

CONUT, controlling nutritional status; AMI, acute myocardial infarct; OR, odds ratio; 95% CI, 95% confidence interval; MACE, major adverse cardiovascular events; STEMI, ST-elevation myocardial infarction; NSTEMI, non-ST elevated myocardial infarction; PCI, percutaneous coronary intervention.

The results of subgroup analysis of mortality with CONUT score as a categorical variable based on follow-up duration, region or country, sample size, age, cutoff values, and treatment methods were all significant (all *p* < 0.05). When the studies were stratified by patient population, CONUT score was significantly predictive for NSTEMI and mixed populations, but not for STEMI patients (Table 2).

Subgroup analysis of stroke using CONUT score as a categorical variable indicated that CONUT score was markedly linked to stroke in AMI patients with a follow-up duration of ≤2 years, in Asian populations, and in those receiving PCI treatment only. However, no significant association was observed in those with a follow-up duration of >2 years, in European populations, or in patients receiving mixed treatments. Additionally, subgroup analyses by sample size and age did not show significant results. We found that *p*-values for heterogeneity for follow-up duration, European populations, sample size ≤1,000, age ≤65 years, and treatment method were all <50%, suggesting that these factors could be potential sources of heterogeneity (Table 3).

**Table 3:** Subgroup analysis of CONUT and stroke and cardiac death in patients with AMI.

Subgroup	Stroke (categorical variable)				Cardiac death (categorical variable)			
	Study	OR [95% CI]	*p*-value	*I* ^2^	Study	OR [95% CI]	*p*-value	*I* ^2^
Total population	6	1.52 [0.98–2.35]	0.06	51%	7	2.81 [1.67–4.73]	0.0001	81%
STEMI	/	/	/	/	2	2.03 [0.52–7.92]	0.31	58%
NSTEMI	/	/	/	/	/	/	/	/
Mixed	/	/	/	/	5	3.09 [1.70–5.62]	0.0002	87%
Follow-up								
>2 years	3	1.16 [0.86–1.56]	0.33	20%	5	2.06 [1.35–3.16]	0.0009	73%
≤2 years	3	2.98 [1.56–5.66]	0.0009	0%	2	11.06 [4.80–25.51]	0.00001	0%
Region								
Asia	1	2.99 [1.43–6.25]	0.004	NA	3	3.87 [0.88–16.94]	0.07	81%
Europe	5	1.21 [0.91–1.62]	0.20	12%	4	2.20 [1.36–3.55]	0.001	78%
America	/	/	/	/	/	/	/	/
No. of patients								
>1,000	4	1.43 [0.88–2.30]	0.15	64%	4	2.93 [1.58–5.42]	0.0006	89%
≤1,000	2	2.94 [0.79–10.97]	0.11	0%	3	2.61 [0.81–8.42]	0.11	45%
Mean/median age								
>65 years	4	1.43 [0.88–2.30]	0.15	64%	4	2.93 [1.58–5.42]	0.0006	89%
≤65 years	2	2.94 [0.79–10.97]	0.11	0%	3	2.61 [0.81–8.42]	0.11	45%
Cut-off								
>3	/	/	/	/	2	2.03 [0.52–7.92]	0.31	58%
≤3	/	/	/	/	2	11.06 [4.80–25.51]	0.00001	0%
Treatment								
PCI only	3	2.98 [1.56–5.66]	0.0009	0%	4	4.35 [1.27–14.89]	0.02	72%
Mixed	3	1.16 [0.86–1.56]	0.33	20%	3	2.09 [1.29–3.38]	0.003	84%

CONUT, controlling nutritional status; AMI, acute myocardial infarct; OR, odds ratio; 95% CI, 95% confidence interval; STEMI, ST-elevation myocardial infarction; NSTEMI, non-ST elevated myocardial infarction; PCI, percutaneous coronary intervention.

Subgroup analysis of cardiac death using CONUT score as a categorical variable unveiled that the CONUT score was significantly predictive for mixed populations, follow-up duration (both >2 years and ≤2 years), European populations, sample size >1,000, age >65 years, cutoff values ≤3, and treatment methods (including PCI only and mixed treatment). No significant association was observed for other subgroups. Additionally, *p*-values for heterogeneity were <50% in subgroups with follow-up duration ≤2 years, sample size ≤1,000, age ≤65 years, and cutoff values ≤3, indicating that these factors could be potential sources of heterogeneity (Table 3).

Subgroup analysis of myocardial reinfarction using CONUT score as a categorical variable unveiled that CONUT score was markedly linked to reinfarction outcomes in AMI patients with a follow-up duration of ≤2 years, sample size >1,000, age ≤65 years, cutoff values ≤3, and in those receiving PCI treatment only. No significant association was observed for other subgroups. Among these, the *p*-values for the heterogeneity for follow-up duration of ≤2 years were <50%, suggesting that it might serve as a source of heterogeneity in this subgroup (Table 4).

**Table 4:** Subgroup analysis of CONUT and myocardial reinfarction in patients with AMI.

Subgroup	Myocardial reinfarction (categorical variable)			
	Study	OR [95% CI]	*p*-value	*I* ^2^
Total population	7	2.21 [1.28–3.83]	0.005	88%
STEMI	3	3.00 [0.74–12.06]	0.12	86%
NSTEMI	/	/	/	/
Mixed	4	1.61 [1.00–2.60]	0.05	82%
Follow-up				
>2 years	5	1.65 [0.96–2.84]	0.07	86%
≤2 years	2	4.61 [2.70–7.84]	0.00001	0%
Region				
Asia	3	3.00 [0.74–12.06]	0.12	86%
Europe	4	1.61 [1.00–2.60]	0.05	82%
America	/	/	/	/
No. of patients				
>1,000	4	1.81 [1.05–3.13]	0.03	89%
≤1,000	3	3.14 [0.56–17.59]	0.19	86%
Mean/median age				
>65 years	3	1.35 [0.88–2.07]	0.16	81%
≤65 years	4	3.49 [1.21–10.03]	0.02	80%
Cut-off				
>3	3	3.00 [0.74–12.06]	0.12	86%
≤3	1	5.56 [1.69–18.27]	0.005	NA
Treatment				
PCI only	4	3.49 [1.21–10.03]	0.02	80%
Mixed	3	1.35 [0.88–2.07]	0.16	81%

CONUT, controlling nutritional status; AMI, acute myocardial infarction; OR, odds ratio; 95% CI, 95% confidence interval; STEMI, ST-elevation myocardial infarction; NSTEMI, non-ST elevated myocardial infarction; PCI, percutaneous coronary intervention.

### 3.7 GRADE assessment

The GRADE system was leveraged to appraise the evidence quality for various outcomes in AMI patients, including MACE, mortality, stroke, cardiac death, myocardial reinfarction, revascularization, ventricular arrhythmias, and atrioventricular block. The evidence quality for the categorical variables of MACE, stroke, revascularization, and ventricular arrhythmias, as well as the continuous variables of mortality, was classified as very low. The evidence quality for the categorical variables of mortality, cardiac death, myocardial reinfarction, and atrioventricular block was rated as low. The evidence quality for continuous variables of MACE was graded as moderate (Supplementary Table S3).

## 4 Discussion

The current meta-analysis demonstrated that CONUT score, based on three common laboratory biomarkers (serum albumin, total cholesterol level, and total peripheral lymphocyte count), was linked to clinical outcomes in AMI patients. A high CONUT score was indicative of unfavorable prognosis in AMI patients and showed a strong correlation with MACE, mortality, cardiac death, myocardial reinfarction, and atrioventricular block. However, it showed no significant association with adverse outcomes such as stroke, revascularization, or ventricular arrhythmias. Sensitivity analysis revealed the instability of analysis results of stroke after AMI, indicating that currently, no sufficient evidence supports a definitive link between the two. Due to a small number of studies on ventricular arrhythmias and atrioventricular block, sensitivity analyses were not performed for these outcomes. Egger’s test suggested publication bias for MACE and mortality as categorical variables, which could potentially affect the GRADE quality rating of the evidence. Further research is warranted for confirmation. The results of the remaining outcome measures appeared to be stable and free from publication bias. Hence, future studies on the prevention, early diagnosis, and intervention of clinical outcomes in AMI patients are needed. The risk of malnutrition is frequently observed among patients with cardiovascular disease, and the significance of the CONUT score in the treatment and prognosis of these patients has been increasingly recognized. A recent meta-analysis demonstrated that the CONUT score is an independent prognostic factor for mortality in stroke patients and is directly associated with the development of disability and infections. Therefore, the CONUT score also holds significant prognostic value for cerebrovascular diseases (35). Thus, CONUT score can be utilized to early detect the risk of malnutrition in hospitalized patients and continuously monitor their nutritional status during treatment. Additionally, it can be utilized to detect the risk of malnutrition in primary care patients (36).

Previous studies on the link between the CONUT score and unfavorable prognosis in CAD patients have been published (19). However, these studies focused primarily on CAD patients and only examined MACE and mortality, without giving due attention to other clinical outcomes. Our study specifically targeted the AMI population, including 15 studies involving a total of 14,302 patients. We not only analyzed the link of the CONUT score to MACE and mortality in AMI patients but also focused on other clinically common prognostic outcomes such as stroke, cardiac death, revascularization, myocardial reinfarction, ventricular arrhythmias, and atrioventricular block. Additionally, we conducted detailed subgroup analyses for outcomes such as MACE, mortality, stroke, cardiac death, and myocardial reinfarction in AMI patients. As far as we know, this is the first meta-analysis specifically elucidating the prognostic significance of the CONUT score in AMI patients.

An array of meta-analyses have demonstrated that CONUT score is also linked to the outcomes of individuals with other cardiovascular diseases. The meta-analysis by Huang et al. (37) with 12,532 patients indicated that CONUT score was markedly linked to all-cause mortality in heart failure (HF) patients. Their results unraveled that this score can be leveraged to appraise the nutritional status in HF. Similarly, Kazemian et al. (38) unveiled that a heightened CONUT score was linked to an elevated 1-year mortality rate in individuals treated with transcatheter aortic valve implantation (TAVI). Likewise, our analysis has unraveled a significant impact of the CONUT score on the prognosis of AMI patients, which is generally consistent with the findings from studies on other cardiovascular diseases.

Given the high heterogeneity in this meta-analysis, we conducted subgroup analysis for MACE, mortality, stroke, cardiac death, and myocardial reinfarction in AMI patients to identify potential sources of heterogeneity. Primarily, subgroup analysis by population, treatment methods, and age was executed to explore the efficiency of the CONUT score for forecasting mortality, cardiac death, and myocardial reinfarction in AMI patients. First, the CONUT score is markedly efficient in forecasting mortality and cardiac death in the NSTEMI and mixed population groups, but not in the STEMI subgroup. A possible explanation is that NSTEMI patients tend to have more complex clinical characteristics compared to STEMI patients, including older age and more comorbidities. Studies have reported that the long-term prognosis of NSTEMI patients generally does not improve to the same extent as that of STEMI patients (33). Second, CONUT score was markedly linked to MACE, stroke, and myocardial reinfarction in AMI patients who received PCI, but no significant effect was noted in the mixed treatment group. This is possibly attributed to the fact that most myocardial infarction patients undergoing PCI are malnourished. Persistent malnutrition can postpone tissue repair and accelerate the development of complications. Additionally, atherosclerosis represents a low-level inflammatory process, which leads to altered metabolism, muscle catabolism, and reduced serum albumin levels (39). Low serum albumin and high C-reactive protein (CRP) levels have been demonstrated to adversely impact the long-term prognosis of individuals receiving PCI (40). An interesting finding was observed in the subgroup analysis by age at 65 years. CONUT score was considerably efficient in forecasting cardiac death in AMI patients aged >65 years, but not in those aged ≤65 years, suggesting a significant link between CONUT score and cardiac death in older AMI patients. This is consistent with a previous finding, that is, older age is generally associated with poorer nutritional status (30). Conversely, the CONUT score was a valuable predictor of myocardial reinfarction in individuals with AMI aged ≤65 years, but not in patients aged >65 years. This indicates that in clinical practice, age ≤65 years could be considered a significant factor for examining the correlation between age and post-AMI myocardial reinfarction. The elevated risk of myocardial reinfarction in younger AMI patients with higher CONUT scores is likely due to lifestyle factors commonly observed in younger patients, such as smoking and poor sleep habits. Short sleep duration often leads to increased fat intake and decreased protein intake (41), contributing to the risk of malnutrition. Additionally, long-term tobacco use can perpetuate inflammation and produce a large number of free radicals, leading to reduced protein synthesis, thereby promoting the development of cardiovascular diseases (42). Additionally, disruptions in circadian rhythms (43) and nicotine intake (44), can lead to endocrine disorders, further exacerbating the activation of systemic immune and inflammatory responses. Coupled with excessive consumption of high-sugar and high-carbohydrate diets (45), these combined factors contribute to the increasing prevalence of cardiovascular diseases among younger populations. However, this is only a hypothesis, and large-scale prospective clinical studies are warranted in the future to corroborate these findings.

The risk of malnutrition is proven to be linked to unfavorable prognosis in individuals with various chronic diseases, such as malignancies (46), peripheral vascular diseases (47), and heart failure (5). Nonetheless, the link between the risk of malnutrition and long-term prognosis in the AMI population remains to be elucidated. Some studies have compared different nutritional scores, including the PNI, Geriatric Nutritional Risk Index (GNRI), and CONUT score, to probe into the most suitable tool for evaluating the prognosis of CAD (18). Nevertheless, there is no consensus about which tool is preferable. PNI is a nutritional evaluation tool computed by lymphocyte counts and serum albumin levels. Nevertheless, the cutoff values for diagnosing malnutrition using PNI vary across studies, which limits its widespread clinical application. Some research has unraveled that PNI may not be a robust tool for forecasting the prognosis of AMI (48). The GNRI is computed based on serum albumin and BMI, but it may understate malnutrition in individuals with normal or high BMI (49). In contrast, the CONUT score comprises serum albumin concentration, total lymphocyte count, and total cholesterol levels. Low levels of these laboratory indexes have been demonstrated to be linked to disease progression and elevated mortality in AMI patients (27, 30, 33).

Albeit the precise mechanisms underlying the link between CONUT score and AMI prognosis have not been fully elucidated, they may be explained as follows: first, albumin reflects systemic inflammation and nutritional status (50). Chronic inflammatory diseases can lead to a reduction in albumin levels (39). The risk of malnutrition is closely linked to increased inflammation, which in turn contributes to a greater burden of atherosclerosis. This interrelationship is recognized as the malnutrition-inflammation-atherosclerosis (MIA) syndrome (51). Thus, controlling inflammation is crucial for reducing the likelihood of developing MACE in patients. Albumin can suppress platelet aggregation by stimulating the formation of the anti-aggregatory agent prostaglandin D2 (PGD2) from cyclic endoperoxides. Additionally, due to elevated concentrations of free lysophosphatidylcholine, hypoalbuminemia can increase blood viscosity and lead to endothelial dysfunction (30). Second, the total peripheral lymphocyte count is indicative of cell-mediated immunity. In acute myocardial infarction, circulating cytokines subdue lymphocyte counts, possibly leading to recurrent myocardial infarction (52). Third, impaired cholesterol homeostasis, a part of the immune response, can intensify the inflammatory processes, leading to atherosclerosis. As per the European Society of Cardiology (ESC) guidelines, in spite of low cholesterol levels, lipid-lowering treatment is required for individuals with NSTEMI (53). However, this recommendation must be interpreted cautiously since cholesterol, as one of the variables in the CONUT score, may lead to an overestimated prevalence of malnutrition in cardiovascular disease patients (54). Multiple studies have unraveled an inverse link between high cholesterol levels and cardiovascular adverse events, a phenomenon referred to as the cholesterol paradox (55). One possible explanation is that low total cholesterol levels are indicative of comorbidities such as cachexia, malnutrition, cancer, and other chronic conditions, which in turn reduce cholesterol levels and negatively impact survival outcomes (56). Consequently, malnutrition considerably impacts the occurrence and progression of AMI. Investigating the mechanisms of malnutrition resulting in AMI is essential for formulating suitable preventive and therapeutic approaches.

Our meta-analysis has several limitations that should be considered. First, since all the eligible studies were retrospective, moreover, certain subgroup analyses had relatively small sample sizes, this study is inevitably impacted by selection bias and confounding factors. Second, individuals with low cholesterol levels may not be treated with aggressive statin therapy, and the CONUT score could be significantly affected by statin or other lipid-lowering treatments. Third, varying degrees of heterogeneity were observed across the incorporated studies, possibly due to distinctions in AMI subtypes, the definition of MACE, or variations in follow-up duration. Hence, the findings of this investigation should be interpreted cautiously. Additionally, considerable heterogeneity was noted in the evaluation of prognostic outcomes across studies. However, subgroup analyses identified some of the sources of this heterogeneity. Lastly, the cutoff values for the CONUT score were not consistent across the included studies, which may also be a major contributor to heterogeneity. Thus, further multicenter prospective trials are needed to validate our meta-analysis findings.

## 5 Conclusion

CONUT score is an important prognostic indicator for poor outcomes in AMI patients, showing a significant association with MACE, mortality, cardiac death, myocardial reinfarction, and atrioventricular block. Given the large number of retrospective studies included, some subgroup analyses had relatively small sample sizes, high heterogeneity, and potential selection bias, larger sample size, multicenter, and prospective clinical studies are desired to determine whether CONUT score can be utilized as a reliable tool for appraising the nutritional status in AMI patients, guiding the development of nutritional interventions for improving their prognosis.

## Data Availability

The original contributions presented in the study are included in the article/Supplementary material, further inquiries can be directed to the corresponding author.
