# Supplementary material for: Association between controlling nutritional status score and the prognosis of patients with acute myocardial infarction: a systematic review and meta-analysis
Source: Front Nutr. 2025 Jan 15;11:1518822. doi: 10.3389/fnut.2024.1518822 (PMC11774715; doi:10.3389/fnut.2024.1518822)
Supplement: Supplementary file 1 [file Supplementary_file_1.docx]

Supplementary Material

**Association between Controlling Nutritional status score and the prognosis of patients with acute myocardial infarction: a systematic review analysis**

Lei Peng^1^, Jian Tang^2^, **Ningjun Zhang**^3^, **Zhongnan Zhang**^1^, **Deqi Wang**^4^, **Youfu He**^5*^

*** Correspondence:** Youfu He, 158301879@qq.com

**Supplementary Table 1.** Search strategy

**PUNMED-209**

((Controlling Nutritional Status) OR (CONUT)) AND (("Myocardial Infarction"[Mesh]) OR (((((((((((((Infarction, Myocardial) OR (Infarctions, Myocardial)) OR (Myocardial Infarctions)) OR (Heart Attack)) OR (Heart Attacks)) OR (Myocardial Infarct)) OR (Infarct, Myocardial)) OR (Infarcts, Myocardial)) OR (Myocardial Infarcts)) OR (Cardiovascular Stroke)) OR (Cardiovascular Strokes)) OR (Stroke, Cardiovascular)) OR (Strokes, Cardiovascular)))

**EMBASE-47**

((Controlling Nutritional Status or CONUT) and (Myocardial Infarction or (Infarction, Myocardial or Infarctions, Myocardial or Myocardial Infarctions or Heart Attack or Heart Attacks or Myocardial Infarct or Infarct, Myocardial or Infarcts, Myocardial or Myocardial Infarcts or Cardiovascular Stroke or Cardiovascular Strokes or Stroke, Cardiovascular or Strokes, Cardiovascular))).af.

**Cochrane-0**

((Controlling Nutritional Status or CONUT) and (Myocardial Infarction or (Infarction, Myocardial or Infarctions, Myocardial or Myocardial Infarctions or Heart Attack or Heart Attacks or Myocardial Infarct or Infarct, Myocardial or Infarcts, Myocardial or Myocardial Infarcts or Cardiovascular Stroke or Cardiovascular Strokes or Stroke, Cardiovascular or Strokes, Cardiovascular))).af.

**Web of Science 162**

((Controlling Nutritional Status) OR (CONUT)) AND ((Myocardial Infarction) OR (((((((((((((Infarction, Myocardial) OR (Infarctions, Myocardial)) OR (Myocardial Infarctions)) OR (Heart Attack)) OR (Heart Attacks)) OR (Myocardial Infarct)) OR (Infarct, Myocardial)) OR (Infarcts, Myocardial)) OR (Myocardial Infarcts)) OR (Cardiovascular Stroke)) OR (Cardiovascular Strokes)) OR (Stroke, Cardiovascular)) OR (Strokes, Cardiovascular))) (Topic)

**Supplementary Table 2.** Quality evaluation of the eligible studies with Newcastle–Ottawa scale.

| **Study** | **Selection** | | | | **Comparability** | | **Outcome** | | |
| --- | --- | --- | --- | --- | --- | --- | --- | --- | --- |
|  | **Representative-ness** | **Selection of**  **non-exposed** | **Ascertainment**  **of exposure** | **Outcome not present at start** | **Comparability on most important factors** | **Comparability on other risk factors** | **Assessment of outcome** | **Long enough follow-up (median≥1 year)** | **Adequacy**  **(completeness) of follow-up** |
| Zengin et al. | * | * | * | * | - | * | * | * | * |
| Roubín et al. | * | * | * | * | - | - | * | * | * |
| Takahashi et al. | * | * | * | * | - | * | * | * | * |
| Lu et al. | * | * | * | * | - | - | * | * | * |
| Kong et al. | * | * | * | * | - | * | * | * | * |
| Czinege et al. | * | * | * | * | - | - | * | * | * |
| RUS et al. | * | * | * | * | - | * | * | * | * |
| Mangalesh et al. | * | * | * | * | - | * | * | * | * |
| Ni et al. | * | * | * | * | - | - | * | * | * |
| Basta et al. | * | * | * | * | - | - | * | * | * |
| Boyraz et al. | * | * | * | * | - | - | * | * | * |
| Chen et al. | * | * | * | * | - | * | * | * | * |
| Deng et al. | * | * | * | * | - | * | * | * | * |
| Kalyoncuo˘ glu et al. | * | * | * | * | - | * | * | * | * |
| Yıldırım et al. | * | * | * | * | - | * | * | * | * |
| *indicates criterion met; - indicates significant of criterion not met. | | | | | | | | | |

**Supplementary Table 3.** GRADE rating of each outcome.

| **No. of studies** | **Outcomes** | **Metrics** | **Estimate** | **95%CI** | **I2; P value** | **Risk of bias** | **Inconsistency** | **Indirectness** | **Imprecision P<0.001** | **Publication bias** | **Plausible confounding** | **Magnitude of effect** | **Dose-response gradient** | **GRADE** |
| --- | --- | --- | --- | --- | --- | --- | --- | --- | --- | --- | --- | --- | --- | --- |
|  |  |  |  |  |  |  |  |  |  |  |  |  |  |  |
| 12 | MACE | OR | 1.75 | 1.42, 2.15 | 88%; P<0.00001 | No serious risk | Serious inconsistency | No serious | No serious | Strongly suspected | Would reduce effect | No | No | Very Low |
|  |  |  |  |  |  |  |  |  |  |  |  |  |  |  |
| 3 | MACE | SMD | 1.02 | 0.78, 1.26 | 21%; P=0.28 | No serious risk | No serious | No serious | No serious | Undetected | Would not reduce effect | No | No | Moderate |
|  |  |  |  |  |  |  |  |  |  |  |  |  |  |  |
| 17 | Mortality | OR | 2.08 | 1.70, 2.55 | 88%; P<0.00001 | No serious risk | Serious inconsistency | No serious | No serious | Strongly suspected | Would reduce effect | Yes | No | Low |
|  |  |  |  |  |  |  |  |  |  |  |  |  |  |  |
| 2 | Mortality | SMD | 1.16 | 0.57-1.74 | 92%; P=0.0003 | No serious risk | Serious inconsistency | No serious | No serious | NA | Would not reduce effect | NO | NO | Very Low |
|  |  |  |  |  |  |  |  |  |  |  |  |  |  |  |
| 6 | Stroke | OR | 1.52 | 0.98,2.35 | 51%;P=0.07 | No serious risk | Serious inconsistency | No serious | serious | Undetected | Would not reduce effect | NO | NO | Very Low |
|  |  |  |  |  |  |  |  |  |  |  |  |  |  |  |
| 7 | Cardiac death | OR | 2.81 | 1.67,4.73 | 81%;P<0.0001 | No serious risk | Serious inconsistency | No serious | No serious | Undetected | Would not reduce effect | Yes | NO | Low |
|  |  |  |  |  |  |  |  |  |  |  |  |  |  |  |
| 7 | Myocardial reinfarction | OR | 2.21 | 1.28,3.83 | 88%;P<0.00001 | No serious risk | Serious inconsistency | No serious | serious | Undetected | Would reduce effect | Yes | NO | Low |
|  |  |  |  |  |  |  |  |  |  |  |  |  |  |  |
| 4 | Vessel revascularization | OR | 2.92 | 0.58,14.79 | 84%;P＝0.00003 | No serious risk | Serious inconsistency | No serious | serious | Undetected | Would not reduce effect | NO | NO | Very Low |
|  |  |  |  |  |  |  |  |  |  |  |  |  |  |  |
| 2 | Ventricular arrhythmias | OR | 2.57 | 0.06,107.21 | 83%;P＝0.01 | No serious risk | Serious inconsistency | No serious | serious | NA | Would not reduce effect | NO | NO | Very Low |
|  |  |  |  |  |  |  |  |  |  |  |  |  |  |  |
| 2 | AV block | OR | 5.21 | 1.83, 14.89 | 0%;P＝0.41 | No serious risk | No serious | No serious | serious | NA | Would not reduce effect | Yes | NO | Low |
|  |  |  |  |  |  |  |  |  |  |  |  |  |  |  |

**Abbreviations:** OR, Odds Ratio; 95%CI, 95% confidence interval; MACE, major adverse cardiovascular events;SMD,standard mean difference; GRADE:Grade of Recommendations Assessment，Development and Evaluation; AV,atrio-ventricular.
